# A Narrative Review of Breastfeeding and Its Correlation With Breast Cancer: Current Understanding and Outcomes

**DOI:** 10.7759/cureus.44081

**Published:** 2023-08-25

**Authors:** Merin Abraham, Muhammad Ali Lak, Danyel Gurz, Freida Oshin Martinez Nolasco, Preethi Kamala Kondraju, Javed Iqbal

**Affiliations:** 1 Department of Internal Medicine, Kasturba Medical College, Manipal, IND; 2 Department of Internal Medicine, Combined Military Hospital, Lahore, PAK; 3 Department of Medicine, Universidad de El Salvador, San Salvador, USA; 4 Department of Internal Medicine, Guntur Medical College, Guntur, IND; 5 Department of Neurosurgery, Mayo Hospital, Lahore, PAK

**Keywords:** oncology, breastfeeding promotion, breast milk, breast feeding, breast cancer

## Abstract

Breastfeeding has been extensively studied in relation to breast cancer risk. The results of the reviewed studies consistently show a decreased risk of breast cancer associated with breastfeeding, especially for 12 months or longer. This protective effect is attributed to hormonal, immunological, and physiological changes during lactation. Breastfeeding also appears to have a greater impact on reducing breast cancer risk in premenopausal women and specific breast cancer subtypes. Encouraging breastfeeding has dual benefits: benefiting infants and reducing breast cancer risk long-term. Healthcare professionals should provide evidence-based guidance on breastfeeding initiation, duration, and exclusivity, while public health policies should support breastfeeding by creating enabling environments. This review examines the existing literature and analyzes the correlation between breastfeeding and breast cancer risk.

## Introduction and background

Breast cancer is the most frequent form of cancer among women [1]. In 2020, there were more than 2.3 million new instances of breast cancer and 685,000 fatalities all over the world [2]. With incidence rates ranging from less than 40 per 100,000 females in some Asian and African nations to over 80 per 100,000 in Australia/New Zealand, Northern America, and portions of Europe, a significant geographic range exists among nations and global regions. Mortality showed a less pronounced geographic variation, although compared to transitioned countries, breast cancer fatalities continue to be disproportionately more prevalent in transitioning nations. Because of population growth and aging alone, the burden of breast cancer is expected to rise to almost 3 million new cases and 1 million deaths annually by 2040 [2].

Numerous risk factors for breast cancer have been studied, and the most prevalent ones are early menarche, late menopause, brief or lifetime breastfeeding duration, late age of first full-term pregnancy, low parity, genetics, and nulliparity [3].

Mortality and incidence of breastfeeding differ across races and countries, reflecting variations in risk factors, healthcare access and systems, genetics, lifestyle and cultural influences, attitudes toward screening and treatment, and other socioeconomic factors. Developed countries, such as the United States, Canada, Australia, and Western European nations, generally have higher breast cancer incidence rates and better survival outcomes due to advanced healthcare systems and robust screening programs [4]. In developing countries, breast cancer is often diagnosed at later stages, leading to higher mortality rates. Limited access to healthcare, low awareness, and cultural taboos surrounding breast health can contribute to this disparity. Some countries, such as India and China, are witnessing a rising burden of breast cancer due to changing lifestyles, urbanization, and improved detection methods [4]. Breast cancer affects individuals worldwide, but there are certain racial and ethnic disparities in its incidence and outcomes. In the United States, breast cancer tends to be more common among white women compared to women of other races [5]. However, African-American women tend to experience higher mortality rates from breast cancer than White women. In some Asian countries, such as China and Japan, breast cancer incidence has traditionally been lower than in Western countries, but rates have been steadily increasing. Hispanic and Native American women in the United States may have lower incidence rates than non-Hispanic White women, but mortality rates are often higher [5]. Efforts to reduce breast cancer mortality and improve outcomes require comprehensive strategies, including early detection, education, improved access to healthcare, and culturally sensitive interventions.

Breastfeeding is the natural process of feeding an infant with breast milk produced by the mother’s mammary glands. It is recommended by healthcare professionals globally as the optimal source of nutrition for infants, especially during the first six months of life. Breast milk provides all the necessary nutrients, antibodies, and immune factors that support the newborn’s growth, development, and overall health. It promotes healthy weight gain, boosts the immune system, helps prevent malnutrition and childhood obesity, and gives protection against various illnesses, including allergies, ear infections, respiratory infections, and gastrointestinal tract infections. Breast milk also contains prebiotics that contribute to the development of healthy gut bacteria and is easily digestible. It, hence, plays a crucial role in reducing the chances of digestive issues such as constipation, diarrhea, and gastroenteritis and improves the infant’s overall metabolic and immune health. The essential fatty acids, growth factors, and hormones in breast milk support brain development and neurodevelopmental outcomes. Breastfeeding has been associated with improved cognitive development and higher IQ scores in children. Breastfeeding has been shown to reduce the risk of breast cancer in mothers. The longer a woman breastfeeds over her lifetime, the greater the reduction in breast cancer risk. This protective effect is attributed to the hormonal changes that occur during lactation, which lead to the differentiation of breast cells and reduction in the number of breast cell divisions, thereby decreasing the likelihood of genetic mutations that may lead to cancer [5]. Additionally, breastfeeding confers long-term protection to the child by reducing their risk of developing breast cancer later in life. Studies have shown that the longer an individual is breastfed as an infant, the lower their risk of developing breast cancer in adulthood [5,6]. This review aims to assess the existing literature and analyze the correlation between breastfeeding and breast cancer risk of the mother.

## Review

Discussion

Breast Milk

Human milk's abundant components confer enduring benefits [7]. Predominantly, extended breastfeeding is linked to lower breast cancer rates [8]. A human milk complex of alpha-lactalbumin and oleic acid triggers cancer cell apoptosis, sparing normal cells [8]. Breast milk's lysozymes inhibit bacterial proliferation, while lactoferrin hinders bacterial growth by sequestering essential iron [9]. Rich in cytokines with diverse properties, breast milk contains elevated interleukins, interferon γ, tumor necrosis factor α, and more [10]. Protease inhibitors like α1-antichymotrypsin and α1-antitrypsin facilitate neonatal intestinal cytokine transmission [11]. Proline-rich polypeptide supports T cell maturation and prevents autoimmune disorders [12]. Casein and its derivatives counter enamel demineralization, averting dental caries [1,12]. Protein hydrolysates alleviate infant colicky pain [2,12].

Breast Cancer

Breast cancer is a prevalent disease that develops and spreads through complex genetic and clinical mechanisms. Age and family history, particularly having a first-degree relative with the disease, increase the risk of breast cancer [13]. There are numerous genetic factors involved with breast cancer. Certain high-risk predisposition alleles that increase the risk of developing breast cancer have been identified. These include mutations in genes BRCA1 and BRCA2, associated with a 50-85% lifetime risk of breast cancer. Mutations in the TP53 gene can lead to Li-Fraumeni syndrome and increase the risk of breast cancer [14,15]. Other genes, such as PTEN [16] (associated with Cowden syndrome), STK11 [17] (associated with Peutz-Jeghers syndrome), neurofibromatosis [18], and CDH-1 (E-cadherin) [19], are also implicated in breast cancer predisposition.

In addition to high-risk genes, moderate-risk genes are associated with breast cancer. These include heterozygous mutations in the ataxia-telangiectasia [14] gene, somatic mutations in the tumor suppressor gene CHEK2 [20], and modifier genes of BRCA1 and BRCA2, such as BRIP1 [21] and PALB2 [22]. Furthermore, genome-wide association studies have found frequent low-risk alleles linked to breast cancer susceptibility, while the clinical implications of these results are still being investigated. Moreover, miRNA dysregulation has also been linked to breast cancer. Specific miRNAs that exhibit abnormally high expression silence tumor-suppressor genes, accelerating the development of tumors [23].

The cancer stem cells (CSCs) hypothesis suggests that a small subset of cells possesses unique characteristics and contributes to cancer initiation, resistance, and relapse. Targeting CSCs is an active area of research, with ongoing clinical trials investigating therapies specifically designed to eliminate CSCs while sparing normal stem cells [24]. Effective diagnosis and therapy of breast cancer depend on understanding the disease’s pathological development. Breast cancer advances through various stages, starting with flat epithelial atypia and progressing through ductal hyperplasia, ductal carcinoma in situ, and ultimately invasive ductal carcinoma. Tumors caused by BRCA1 mutations typically exhibit a basal-like phenotype with a high histologic grade. These tumors usually do not express the estrogen receptor, progesterone receptor, or Her2/neu, making them known as triple-negative tumors [25] and are usually more aggressive.

The Correlation Between Breastfeeding and Breast Cancer Risk: Current Evidence

The study on breastfeeding and its association with breast cancer risk yielded noteworthy findings. The results demonstrated a significant inverse relationship between the risk of breast cancer and the duration of breastfeeding in premenopausal women. Statistical analysis indicated a consistent decrease in risk with longer breastfeeding duration, with a significant linear trend observed (p-value of linear trend, 0.02) [26]. in particular, breastfeeding showed a positive impact on certain types of breast cancer, including BRCA-1 [27], HER2+ [28,29], and receptor-negative breast cancers [28,30-34]. However, minimal to no change was observed in receptor-positive breast cancers [35].

In a comparative study, researchers examined two groups of women: those who exclusively breastfed for over 18 months and those who breastfed but never exclusively. The study indicated a potential protective effect associated with extended exclusive breastfeeding. Although the results did not achieve statistical significance, the findings revealed that women who exclusively breastfed had a lower adjusted risk of developing premenopausal breast cancer [36]. Furthermore, among women with a first-degree relative who had breast cancer, ever having breastfed was associated with a lower incidence (HR of 0.41, 95% CI, 0.22-0.75) of breast cancer than never. This finding suggests that there is a potential protective effect of breastfeeding in this specific population [36].

HER2: Regarding HER2+ breast cancer, conflicting findings have been reported in studies investigating the association between pregnancy and HER2+ breast cancer risk. However, consistent evidence indicates that breastfeeding is associated with a lower risk of HER2+ breast cancer [28,37]. The existing evidence on the relationship between breastfeeding and luminal B breast cancer risk is primarily based on larger prior studies, which do not demonstrate a significant association [37-40]. However, a study (N=476) reported a reduced risk of luminal B breast cancer among women who breastfed [OR=1.89 (95% CI=1.22, 2.92)], although further research is needed to confirm this association [29].

HR+: Regarding breast cancer subtypes, cohort studies generally indicate no significant links between breastfeeding and ER+/PR+ or ER+ and/or PR+ breast cancers. Nonetheless, in a subset of these studies, one or two out of four and seven studies, respectively, revealed an inverse correlation [35]. In the context of triple-negative breast cancer (TNBC), a background of breastfeeding was associated with reduced risk (OR = 0.09, 95% CI = 0.005-0.54). This risk reduction was particularly pronounced with extended lifetime breastfeeding [31]. Moreover, a borderline decrease in TNBC risk was observed for breastfeeding periods lasting 24 months or more [32].

BRCA mutations: For women with BRCA1 (n=685) mutations, breastfeeding for more than one year was associated with a 22-50% reduced risk of breast cancer compared to those women who never breastfed (OR=0.55, 95% CI=0.38 to 0.80; P=0.001) [27]. The risk reduction was even greater for longer durations (more than a year) of breastfeeding [3]. However, among women carrying a deleterious BRCA2 (n=280) mutation, breastfeeding did not show an association with a reduced risk of breast cancer (OR=0.95, 95% CI=0.56 to 1.59; P=0.83) [27].

These findings highlight the potential protective effect of breastfeeding in decreasing the risk of breast cancer, particularly in specific subtypes and populations. Further research is necessary to understand the underlying mechanisms better and establish comprehensive recommendations regarding breastfeeding practices for breast cancer prevention.

How Breastfeeding May Lower Breast Cancer Risk: Mechanisms and Theories

By the end of pregnancy, the mammary glands have an outstanding share of well-differentiated secretory units than those of a nullipara. Epithelial cells of these secretory units have a longer cell cycle and possess a more efficient DNA excision repair capacity rendering it unscathed by the effects of genetic mutations [41]. Moreover, physical changes in the epithelial cells of the mammary ducts, including extended terminal differentiation induced by lactation, also decrease breast cancer risk by making the breast tissue more resistant to carcinogenesis [42].

Lactation also causes long-term endogenous hormonal changes, possibly by reducing estrogen and increasing prolactin production, which may decrease a woman’s cumulative exposure to estrogen, thereby inhibiting the initiation or growth of breast cancer cells [43,44]. The effects of breastfeeding may be attributed to its role in delaying the re-establishment of ovulation [45]. It causes a reduction in lifetime exposure to the mitogenic effect of estrogens, thus obstructing the process of ovulation [46]. The secretion of carcinogens from human milk and the exfoliation of the breast cells during lactation assist in destroying the damaged DNA cells, which helps in diminishing the responsiveness to mutations [47]. Furthermore, serum insulin concentrations are minimized by expressing human milk and breastfeeding. Studies have confirmed that proliferation and antiapoptosis effects in breast tissue are correlated with persistent high serum concentrations of insulin, which can raise serum concentrations of IGF-1 (insulin-like growth factor) [48].

One of the primary components of human milk postulated to affect cancer risk is alpha-lactalbumin. HAMLET (a human milk complex of alpha-lactalbumin and oleic acid) can induce tumor cell death. HAMLET induces apoptosis only in tumor cells, while normal differentiated cells resist its effects. Therefore, HAMLET may provide safe and effective protection against the development of breast cancer [5].

Calcium and the presence of breast fluid help reduce the number of cells on the inner surface of the breast by promoting cell growth and differentiation. During breastfeeding, calcium and breast fluid remove breast CSCs [49].

There is also evidence from human cell culture systems that BRCA1 suppresses estrogen-mediated breast cell proliferation [50]. Individuals with low levels of BRCA1 may have increased breast epithelial cell proliferation in response to the increased estrogen exposure during pregnancy [48].

Breastfeeding could drive terminal differentiation of breast ductal cells via increased production of the GATA family of transcription factors, specifically GATA-3, which has been shown to actively maintain the differentiated luminal epithelium actively; thus, conferring protection from aberrant transcription changes, GATA-3 may play a causal role in the loss of tumor differentiation and malignant conversion in breast cancer. The loss of GATA-3 in the mammary gland causes luminal cell proliferation and basement-membrane detachment [51].

Inhibition of exosomes containing miR-29s in primary bovine mammary epithelial cells results in global DNA hypermethylation (gene silencing) and increases promoter methylation of several lactation-related genes, including Elf5 [52]. Since milk exosomes can be taken up by cultured mammary ductal epithelial cells and remain functional [53], it is conceivable that decreased exposure of mammary epithelial cells to milk exosomes in vivo could result in increased expression of DMNTs and consequent aberrant methylation, which leads to cancer [54].

A meta-analysis showed that there is a protective effect of breastfeeding against hormone receptor-negative breast cancers, which are more common in younger women and generally have a poorer prognosis than other breast cancer subtypes [53]. Breastfeeding’s protective effect on ER−/PR− and TNBC subtypes needs further investigation. This protective effect may be partly due to alterations in hormones other than estrogen and progesterone, such as androgens, which can suppress cell proliferation in ER+ tumors but promote tumorigenesis in ER− tumors [48]. The mechanisms of action of parity and breastfeeding’s separate and additive protective effects are likely to work through their effects on the molecular maturation and the complete involution of the terminal ductal units. These milk-making cells confer resistance to carcinogenesis [55].

Epigenetic reprogramming could occur by lack of breastfeeding and result in aberrant DNA methylation of FOXA1 and other genes in breast luminal progenitor cells [56]. If terminal differentiation is blocked, for example, by methylation-associated silencing of FOXA1 due to abrupt involution brought about by shortened or a lack of breastfeeding, this putative “seek and destroy” mechanism may be impaired, allowing these cells to escape immune surveillance and destruction and survive as potential precursors to ER− breast cancer [54].

The Role of Duration and Frequency of Breastfeeding in Breast Cancer Prevention

LncRNAs usually have regulatory roles in multiple processes, including cell differentiation, proliferation, migration, and the cell cycle. A recent study identified upstream Eleanor (u-Eleanor), a novel lncRNA with key functions in breast cancer [57-59].

LncRNAs, u-Eleanor, and HOTAIR are associated with hormone-dependent reproductive risk factors in breast neoplasms [60]. A previous T-test revealed that women without a lactation history had a higher level of u-Eleanor expression than those with a breastfeeding history. Furthermore, as the duration of lactation decreases, the expression of u-Eleanor increases [1,60]. In the same fashion, previous ANOVA demonstrated an increased level of u-Eleanorin in women with a brief duration of breastfeeding (one to six months) compared to those with a longer term of breastfeeding (greater than 24 months) [2,60].

The terminally differentiated cells would be killed by the inflammatory environment associated with the involution of the breast tissue to a pre-pregnancy stage, typically induced by prolonged lactation periods. In contrast, forced involution by a lack of or brief duration of lactation may result in an inflammatory environment that promotes the survival and growth of less differentiated cells and the formation of aggressive TNBCs and PACS [61-63].

Another study by the Journal of Human Lactation revealed that the risk for breast neoplasm is reduced in women who exclusively breastfeed; the breast cancer risk is reduced further if lactation is continued for at least one year [64]. Any duration of lactation was associated with a lower risk of TNBC, and the odds of this phenotype decreased with increasing duration [65].

Breastfeeding and Breast Cancer Outcomes

Breast cancer treatment encompasses a range of therapeutic approaches, including surgical intervention, radiotherapy, and adjuvant drug therapy. Surgical options such as breast-conserving surgery have demonstrated efficacy in removing tumors while preserving the integrity of the breast [66]. However, breast conservative surgery may result in cosmetic alterations, and adjuvant drug therapy can induce amenorrhea [67,68]. Conversely, breastfeeding is widely recognized for its beneficial effects on infants and mothers [69].

The timing of breastfeeding during a breast cancer diagnosis impacts the survival rate. Breastfeeding near diagnosis is linked to lower survival, while breastfeeding improves survival outcomes after diagnosis. Whiteman et al. discovered heightened mortality in women aged 20-45 who gave birth within 12 months prior but no significant association between breastfeeding and prognosis [70]. Similarly, studies examining breastfeeding in women with a history of breast cancer have yielded promising findings. In a comparative analysis involving 94 women, breastfeeding was linked to a lower relapse rate (3%) compared to non-breastfeeding (24%) and unknown lactation status (10%). These results substantiate the safety of breastfeeding in this specific population and indicate no detrimental impact on breast cancer outcomes [71,72]. However, in a study involving 341 women aged 25-74, exploring the relationship between breastfeeding and breast cancer mortality, it was observed that women who breastfed for less than six months faced a significantly elevated risk of breast cancer mortality compared to those who breastfed for a longer duration, 95% CI (HR 2.74; CI 1.41- 5.35), indicating a statistically significant association (p<0.001) [73]. These findings align with a previous prospective study conducted by Kwan et al., which involved two cohorts of women with breast cancer (1636 participants) and demonstrated a strong correlation between breastfeeding for six months or more and a reduced risk of cancer recurrence and breast cancer-related mortality, particularly in the luminal A subtype [30].

Breastfeeding and Breast Cancer Risk in Specific Populations: Age, Ethnicity, and Genetics

Many factors influence infant feeding practices around the world. These include but are not limited to racial and ethnic differences, the mother’s age, maternal knowledge, genetic factors, socioeconomic status, and efficacy in infant feeding [74]. Other risk factors include age >50 and a family history of breast cancer among first-degree relatives <50 [75,76].

Among U.S. women, breast cancer is the most diagnosed cancer. It is typical for women in developed countries to breastfeed for a shorter lifetime duration, contributing to the high incidence of breast cancer in these countries [77]. There are generally poorer breastfeeding practices among women of color than among White women [78]. A study was conducted in Los Angeles comparing 974 post-menopausal women who were recently diagnosed with breast cancer and 973 age and parity-matched controls. It showed that women who breastfed for >/=16 months had lesser odds of developing breast cancer after menopause than women who breastfed for a shorter duration or did not breastfeed at all [79].

Compared to White women, Black women in the United States have lower rates of breastfeeding and nearly two times the rates of more aggressive forms of breast cancer. In general, there is a lack of social and cultural acceptance in their communities, and the support from the healthcare community is also low. These factors and unsupportive work environments magnify their challenges [78]. These women also had limited knowledge regarding the possible association between breastfeeding and the reduction of risk of breast cancer. Another study concluded that Black and Hispanic women are least likely to breastfeed and are more likely to die from breast cancer [80]. Compared to Black women in the United States, a case-control study on African women in Nigeria showed that parity and breastfeeding protected against breast cancer. About 819 breast cancer cases and 569 community controls were interviewed between 1998 and 2006. Compared with women with menarcheal age <17 years, the adjusted OR for women with menarcheal age >or=17 years was 0.72 (95% CI: 0.54-0.95, P=0.02). Parity was negatively associated with risk (P-trend=0.02). Importantly, breast cancer risk decreased by 7% for every 12 months of breastfeeding (P-trend=0.005) [4].

Similarly, a study done in Tunisia examining the association between breastfeeding and the risk of breast cancer reported an inverse association between the two. The mean duration of breastfeeding per child was significantly associated with a reduced risk of breast cancer for women who breastfed for >24 months per child. The OR was 0.46 (95% CI, 0.28-0.76 (p=0.01)) when compared to those who breastfed for <6 months [81].

Mexico has one of the lowest rates of breastfeeding worldwide. A study done in Mexico concluded that there is a high burden of breast cancer due to poor breastfeeding practices. This burden includes morbidity, premature mortality, and economic costs to the health sector and society [82].

Compared to the more developed Western countries, fewer studies have been done in developing Asian countries. Developed Asian countries show similar results as most developed countries in the Western world. A case-control study of breast cancer risk factors in 7,663 women in Selangor, Malaysia, between October 2022 and December 2016 identified longer breastfeeding duration, soy intake, and physical activity as modifiable risk factors for the development of breast cancer [83].

In Asia, a case-control study was conducted among Sri Lankan women aged 30-64, which included 100 cases of histologically confirmed breast cancer and 203 age and parity-matched controls. It showed that women who breastfed >/=24 months during their lifetime had a significantly lower risk of developing breast cancer than those who breastfed for <24 months. Compared to women who breastfed for 0-11 months in their lifetime, there was a 66.3% reduction in breast cancer risk in women who breastfed for 12-23 months, 87.4% reduction in 24-35 months and 94% reduction in 36-47 months categories. This study concluded that prolonged breastfeeding significantly reduces the risk of breast cancer and offers a protective effect [84].

Among Israeli women, investigators noted that breast cancer was more common in nulliparous women and women who did not have a history of breastfeeding. The short duration of lifetime breastfeeding, late age at first breastfeeding, and experience of insufficient milk were found to increase breast cancer risk [85]. Investigators who conducted a study among Iranian women recommended a minimum breastfeeding duration of 18 months, with the best results being achieved with 24 months of breastfeeding. They also suggested that parity of 1-3 protects against breast carcinoma [86]. In Germany, investigators who studied the association between breastfeeding and breast cancer risk reduction by age 50 among women showed a positive association. Even among women with a first-degree family history of breast cancer, they noted a greater reduction in risk with each extra month of breastfeeding [87].

While studies across a majority of the countries concluded that breastfeeding contributes to reducing the risk of breast cancer, some studies found no association or a negative association. A cohort study among Icelandic women indicated a negative association between breastfeeding and breast cancer. However, only two cohort studies have been done there to check the correlation, and the results of these two studies are inconsistent [88]. Another study done in South Brazil concluded that breastfeeding did not have a protective effect against breast cancer [89]. These inconsistencies reiterate that more studies must be done across developing countries.

Challenges and limitations

Breast cancer represents a significant global challenge and an urgent global priority as the burden of the disease is increasing globally. The incidence and mortality are seen more in underserved populations due to late diagnosis [90,91]. Even screening programs require high investments [92]. Discrepancies found within the same country by fragmented healthcare systems result in unequal access in different populations leading to different outcomes [91,92]. Ethical and social implications and patient education also affect the outcomes [93,94].

Breastfeeding is particularly interesting for breast cancer prevention because it is a modifiable risk factor. Breastfeeding reduces breast cancer risk and provides other health benefits to the mother, including reduced risk for endometrial (uterine) cancer, ovarian cancer, hypertension, and diabetes [95].

Breastfeeding problems are common and lead to early cessation [96]. The challenges to the practice of exclusive breastfeeding include problems with latching and pain, infectious problems like mastitis [97], cracked or sore nipples, breast engorgement, insufficient breast milk production, the discomfort of breastfeeding in public, insufficient breastfeeding support from society and healthcare providers, short maternity leave periods, and emotional stress [98,99]. Even marketing breast milk substitutes continues to undermine efforts to improve breastfeeding rates and duration worldwide [100].

Breastfeeding promotion

Breastfeeding has known protective effects against breast cancer, but it is unclear how aware women are of this benefit [101]. The WHO recommends that breastfeeding be initiated within one hour of birth. Infants feed on only breast milk for the first six months of life. Infants continue breastfeeding until at least two years old, and infants are introduced to adequate, safe, and complementary foods at six months [102].

Effective support techniques for breastfeeding include assistance from healthcare professionals like nurses, physicians, and midwives, along with regularly scheduled visits. Targeted support directed toward specific groups of people can also be beneficial [103]. The discussion of breastfeeding during early prenatal care can positively affect a woman’s likelihood of breastfeeding her child. During regular checkups, healthcare providers can discuss the benefits of breastfeeding, potentially influencing women to breastfeed longer than they initially planned [104].

Worldwide efforts are also being made to promote breastfeeding. The Baby-Friendly Hospital Initiative, supported by WHO and UNICEF, encourages hospitals to follow the “Ten Steps to Successful Breastfeeding” and implement the International Code of Marketing of Breast-milk Substitutes. These measures promote breastfeeding and provide support for new mothers. World Breastfeeding Week, an international initiative, also aims to promote exclusive breastfeeding of newborns for the first six months of life. WHO and UNICEF recommend initiating breastfeeding to infants within the first hour of birth and exclusively breastfeeding for the six months of a baby’s life without any other foods or liquids. It provides essential nutrients and health benefits for the baby [104].

## Conclusions

Breast cancer is a significant health concern affecting women worldwide. Breastfeeding, a modifiable risk factor for breast cancer, is inversely related to breast cancer in premenopausal women. The protective effect is increased with increased duration and frequency of breastfeeding. Its protective effect is attributed to hormonal, immunological, and physiological changes during lactation. There is cellular differentiation of breast tissue and a reduction in the lifetime number of ovulatory cycles, thus reducing exposure to estrogen hormone. Therefore, breastfeeding is associated with a decreased risk of breast cancer, and encouraging mothers to breastfeed has dual benefits: benefiting infants and reducing breast cancer risk long-term to mothers.
